# INVESTIGATING THE IMPACTS OF METFORMIN ON AGING AND LIFESPAN IN DIVERSITY OUTBRED MICE

**DOI:** 10.1093/geroni/igad104.3443

**Published:** 2023-12-21

**Authors:** Rong Yuan, Yun Zhu, Monica Frungieri, Michal Masternak, Andrzej Bartke

**Affiliations:** Southern Illinois University School of Medicine School of Medicine, Springfield, Illinois, United States; Southern Illinois University School of Medicine School of Medicine, Springfield, Illinois, United States; Instituto de Biología y Medicina Experimental, Buenos Aires, Buenos Aires, Argentina; University of Central Florida College of Medicine, Orlando, Florida, United States; Southern Illinois University School of Medicine School of Medicine, Springfield, Illinois, United States

## Abstract

Metformin, a type 2 diabetes treatment, has profound effects on regulating development, metabolism, and aging acting via the mTOR/autophagy pathway. Nevertheless, its potential to extend lifespan and counteract aging remains an open question. Previous research highlighted the intricate interplay between genetic background, gender, and treatment commencement age in shaping outcomes. To examine the long-term effect of metformin on aging and lifespan, this project utilizes the diversity outbred (DO) mice, initially derived from the interbreeding of eight inbred strains, effectively emulating the intricate genetic complexity found in humans. Initiated at 2 months old, DO mice were subjected to metformin-infused food [0.1%, w/w] (n=15 and 14, females and males respectively) or a metformin-free counterpart (n=10 for both sexes). Starting from 12 months of age, a statistically significant increase in male body weight was observed within the metformin-treated group, while no significant effect was found in females. Indirect calorimetry assays revealed a significantly elevated respiratory ratio in both sexes of metformin-treated mice. Surprisingly, at the age of 12 months, metformin treatment significantly impaired glucose tolerance in females, whereas in males, pyruvate and insulin tolerance exhibited significant reductions. After the first submission deadline, by 16 months of age, two females died in each of the treatment and control groups. Notably, the metformin-treated male cohort lost four individuals, but no mortality in the control male cohort (P=0.07, log-rank test). These preliminary findings underscore a potential deleterious impact of metformin on metabolic traits and lifespan when initiated from a young age, particularly in male mice.

